# Challenges to driver licensing participation for Aboriginal people in Australia: a systematic review of the literature

**DOI:** 10.1186/s12939-016-0422-9

**Published:** 2016-08-31

**Authors:** Patricia Cullen, Kathleen Clapham, Kate Hunter, Rebekah Treacy, Rebecca Ivers

**Affiliations:** 1 The George Institute for Global Health, Level 3, 50 Bridge Street, Sydney, NSW 2000 Australia; 2 School of Public Health, Sydney Medical School, Edward Ford Building, The University of Sydney, Sydney, NSW 2006 Australia; 3 Australian Health Services Research Institute, University of Wollongong, Building 234, Innovation Campus, Wollongong, NSW 2522 Australia; 4 The Poche Centre for Indigenous Health, The University of Sydney, Edward Ford Building, Sydney, NSW 2006 Australia

**Keywords:** Aboriginal, Driver licensing, Transport disadvantage, Transport injury, Social inclusion

## Abstract

**Introduction:**

Aboriginal and Torres Strait Islander people are overrepresented in transport-related morbidity and mortality. Low rates of licensure in Aboriginal communities and households have been identified as a contributor to high rates of unlicensed driving. There is increasing recognition that Aboriginal people experience challenges and adversity in attaining a licence. This systematic review aims to identify the barriers to licence participation among Aboriginal people in Australia.

**Method:**

A systematic search of electronic databases and purposive sampling of grey literature was conducted, two authors independently assessed publications for eligibility for inclusion.

**Results:**

Twelve publications were included in this review, of which there were 11 reporting primary research (qualitative and mixed methods) and a practitioner report. Barriers identified were categorised as individual and family barriers or systemic barriers relating to the justice system, graduated driver licensing (GDL) and service provision. A model is presented that depicts the barriers within a cycle of licensing adversity.

**Discussion:**

There is an endemic lack of licensing access for Aboriginal people that relates to financial hardship, unmet cultural needs and an inequitable system. This review recommends targeting change at the systemic level, including a review of proof of identification and fines enforcement policy, diversionary programs and increased provision for people experiencing financial hardship.

**Conclusion:**

This review positions licensing within the context of barriers to social inclusion that Aboriginal people frequently encounter. Equitable access to licensing urgently requires policy reform and service provision that is inclusive, responsive to the cultural needs of Aboriginal people and accessible to regional and remote communities.

**Electronic supplementary material:**

The online version of this article (doi:10.1186/s12939-016-0422-9) contains supplementary material, which is available to authorized users.

## Introduction

Transport injuries are a leading cause of morbidity and mortality among Aboriginal and Torres Strait Islander people in Australia [1]. Further, transport injury disproportionately impacts the Aboriginal population with a mortality rate almost three times higher than the non-Aboriginal population [2]. This disparity indicates that strategies for reducing transport-injury have not been as effective in Aboriginal communities. Risk factors for transport injury have been identified in Aboriginal communities including remoteness, non-use of seatbelts, alcohol use, vehicle overcrowding and unlicensed driving [3].

Unlicensed driving is considered prevalent in Aboriginal communities and relates to estimated low levels of licence participation among eligible Aboriginal people [3]. In New South Wales (NSW), it is estimated that Aboriginal people comprise 0.5 % of licensed drivers despite comprising 2 % of the eligible population [4]. While licence participation rates have not been quantified in other Australian jurisdictions, there is increasing recognition that Aboriginal people are being underserviced by the licensing system across Australia; however, there is limited empirical research that investigates the specific barriers to licensing that is impeding Aboriginal people from accessing a driver licence.

In all Australian jurisdictions, attaining a driver licence requires progression through a Graduated Driver Licensing (GDL) Scheme. GDL, considered to be a highly successful road safety strategy, was first introduced in NSW, and all Australian jurisdictions have since introduced GDL [5–8]. The components of GDL vary between jurisdictions but typically include the following: 1) computer based testing procedures to attain a Learner driver licence; 2) minimum time period on a Learner licence; 3) minimum number of supervised driving hours to be eligible to apply for a provisional licence test; 4) passing a vehicle on road test to attain a provisional driver licence and drive unsupervised.

In road safety terms, the efficacy of GDL is generally well accepted, however there are mounting concerns that this system is not equitably accessible and may inadvertently disadvantage vulnerable groups in accessing a licence [6, 9, 10]. Additionally, a relationship between licensing and increased contact with the justice system has been identified as a likely barrier to licence participation [4, 6, 11]. Further, remoteness from service provision, financial hardship and unmet cultural needs are known to adversely impact access to other government and health services in Aboriginal communities; however it is not known how these factors interact with the licensing system.

There is an increasing recognition of the association between social capital and health disparity among the Aboriginal population in Australia [12]. Despite this, driver licensing is frequently overlooked as a means to impact health and social inclusion. To better understand the factors that may be preventing Aboriginal people from accessing a licence, we aimed to draw together literature from diverse methodologies, sources and jurisdictions. Accordingly, we conducted a systematic review of the literature to determine: What are the barriers to licensing for Aboriginal people across jurisdictions in Australia?

## Methods

### Inclusion criteria

We included publications from peer-reviewed and non-peer reviewed literature with no set restrictions on the type, design or methods. To be included in the final review, publications had to be published from the year 2000 onwards, based within the Australian context and Aboriginal and/or Torres Strait Islander populations. Only publications that were specifically related to barriers to driver licensing were included in the final review.

### Search strategy and publication selection

A systematic search of electronic databases was conducted including Medline, ATSIHealth (via Informit Online), PubMed, Scopus and CINAHL. Search terms included combinations of the following: Indigenous, Aborigin*, licen*, unlicen*, drive*, driving, road*, transport*safe*, program*, injur*, crash*, accident*, disadvantag* (Additional file 1: Table S1). The grey literature was also purposively searched including Indigenous HealthInfoNet, Google Scholar and relevant government department websites. All searches were conducted by the first author (PC) in September 2015 and repeated in January 2016.

**Fig. 1:**
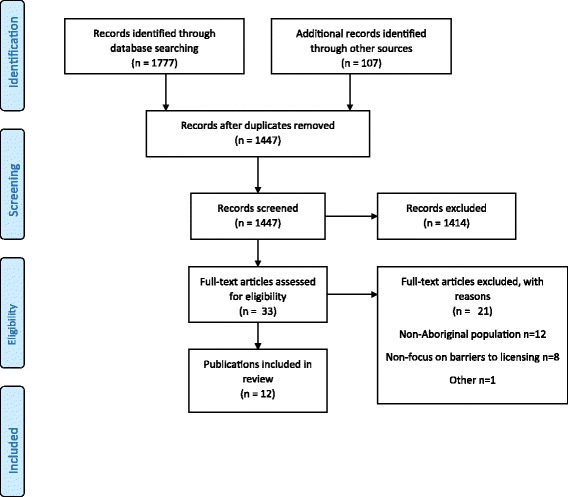
Search and publication selection

After duplicates were removed, the retrieved publications were independently screened for relevance by title and abstract by two authors PC and RT. Publications selected as relevant, were then independently reviewed against the inclusion criteria by PC and RT, any disagreements were resolved by consensus-based discussion. The publication selection process is summarised in a standardised flow diagram (Fig. 1).

### Quality appraisal and analysis

Publications presenting primary research that were appropriate for quality appraisal were assessed using the Mixed Methods Appraisal Tool (MMAT) – Version 2011 [13]. The MMAT critical appraisal tool was selected as it permits researchers to review studies of diverse designs; it has been found to be efficient, reliable and has demonstrated content validity [14–16]. The MMAT allows for concomitant appraisal of qualitative, quantitative and/or mixed methods studies. Relevant data from the included publications was extracted and summarised by PC (Table 1).

**Table 1:** Summary of retained records

Publication	Description	Purpose	Quality^a^	Barriers identified
Anthony and Blagg [23]	Mixed methods research report (Analysis of licensing, offence and injury data, interviews *n* = 16 and focus groups)	Report to the Criminology Research Advisory Council exploring licensing, unauthorised driving and outlining alternate pathways to regulating minor driving offences in remote Indigenous communities	***	Service provision in remote communities, provision of licensing services by police, lack of roadworthy cars, licensing sanctions due to fine default, proof of identification, lack of culturally appropriate support programs
Clapham, Khavarpour [20]	Mixed methods program evaluation (5 focus groups *n* = 55, 23 interviews, program data, offence data)	Evaluation of ‘On the Road’ driver education program targeting Aboriginal people on far North Coast of NSW	*	Proof of identification, literacy (and computer skills), licensing sanctions due to fine default, meeting requirements of GDL (supervised driving practice)
Edmonston, Rumble [27]	Qualitative formative research (interviews *n* = 50 with Indigenous licensing offenders and focus groups with community members and interagency groups)	Describes methodology and preliminary results to inform development of Queensland Indigenous licensing project	***	Service provision in remote communities, provision of licensing services by police, literacy, language, relevance of tests in remote locations (ie city road rules not suited to remote contexts), lack of local driving instructors, proof of identity, access to roadworthy vehicles, lack of understanding and awareness of licensing regulations (e.g. cannot drive unsupervised as a Learner driver)
Elliot and Shananhan Research [24]	Mixed methods research report (15 focus groups and *n* = 300 structured interview/surveys)	Report to Roads and Traffic Authority of NSW to quantify and identify licensing issues for Aboriginal people, direct policy and make recommendations for service delivery and monitoring effectiveness	***	Prohibitive costs associated with licensing, licensing sanctions due to fine default, literacy, “shame”, fear of failure and discomfort with service providers e.g. Roads and Maritime Services. Survey results reinforced costs and fine default as main deterrent, and also health problems, older age and literacy
Helps, Moller [28]	Literature review and qualitative research report (series of discussion forums, 3 focus groups *n* = 30, interviews/case studies *n* = 10)	Explore the issues around safe and accessible transport and for Aboriginal people in South Australia. Focus areas were: driver licensing, seat restraints, transport issues relating to health and disability.	***	Language, literacy, provision of licensing services by police, prohibitive costs associated with licensing, apprehension toward service providers (local licensing authorities), sanctions due to fine default.
Ivers, Hunter [29]	Synthesis of literature, key informant perspectives and audit of programs	Report to the National Road Safety Council to explore key issues relating to road safety and driver licensing for Aboriginal people in Australia	N/A	Proof of identification, service provision in remote communities, prohibitive costs associated with licensing, literacy, meeting the requirements of GDL (lack of supervisory drivers) and sanctions due to fine default
Ivers, Lyford [25]	Mixed methods pilot study (3 focus groups *n* = 17 and survey *n* = 27)	Pilot recruitment and data collection methods, and to identify community road safety concerns and priorities	***	Prohibitive costs associated with licencing, “shame” and fear of failure, proof of identification, literacy, sanctions due to fine default, meeting the requirements of GDL (lack of supervisory drivers for 120 h)
Job and Bin-Sallik [21]	Program Evaluation	Development and implementation of the DriveSafe NT Remote mobile licensing support program	N/A	Proof of identity, literacy, language, remoteness and lack of access to training services, limited access to legal vehicles for and supervisory drivers for driving practice
NSW Auditor General [26]	Mixed methods research report (analysis of NSW licensing and offence data, key informant perspectives and audit of programs)	Review barriers to licensing for Aboriginal people in NSW, and investigate government responses and the effectiveness of actions to prevent or reduce barriers	N/A	Meeting the requirements of the GDL was cited as the main barrier. More specifically the lack of access to appropriate supervisory drivers and literacy, access to licensing services (e.g. to practice the knowledge test), prohibitive costs associated with licensing, proof of identity, sanctions due to fine default, lack of diversionary options, understanding legal processes and requirements (e.g. court attendance)
Rumble and Fox [22]	Program Evaluation	Development and implementation of the Queensland Aboriginal peoples and Torres strait Islander peoples driver licensing program	N/A	Proof of identification, literacy, service provision in remote locations, fear of failure, fear of authorising agencies (e.g. police), sanctions due to fine default
Somssich [31]	Practitioner report	Review of the impact of policy on driver licensing and training programs in remote Aboriginal communities in the Northern Territory	N/A	Changes to policy including increased costs and procedures for ensuring proof of identification, which can be problematic in remote communities and for people who have dual names, low income and low literacy. Meeting requirements of GDL (mandatory time periods on Learner licence), lack of local initiatives
Williamson, Thompson [30]	Synthesis of literature, key informant perspectives and audit of programs	Report to South Australia Health to identify barriers and facilitators to driver licensing for Aboriginal people in SA. Describe the relationship between licensing and contact with the justice system and identify initiatives assisting people to overcome barriers	N/A	Prohibitive costs associated with licensing, access to roadworthy vehicle, sanctions due to fine default, meeting the requirements of GDL (access to appropriate supervisory drivers), service provision in remote communities (lack of culturally appropriate service provision, lack of driving instructors, remoteness from licensing authorities, reluctance to engage with service providers (police, local licensing authorities), lack of diversionary programs for offenders, proof of identity, confidence, literacy, health concerns

^a^MMAT scores can range from 25-100 % as follows: 25 % (*), 50 % (**), 75 % (***), 100 % (****)

The data from the selected publications was analysed using a narrative synthesis approach that is appropriate for mixed methods sources [17]. The narrative synthesis was informed by the social ecology model, which has been used to explore inequities that underlie health disparities [18]. The social ecological approach asserts that health is a function of the interrelationship between individual, interpersonal, community, socio-political and environmental influences; this model is inherently suited to exploring complex health and equity issues that are diverse and multi-factorial [19]. For the purposes of this review, barriers were categorised as either systemic or individual and family barriers, however due to the complex nature of the licensing adversity there was some interplay between the categories.

## Results

The search of electronic databases returned 1777 records, and from other searches 107 records were identified; the selection and exclusion process is detailed in Fig. 1. After removing duplicates and screening for relevance, 33 records were retained for eligibility assessment, of which 12 met the inclusion criteria; the reasons for exclusion are outlined in Additional file 1: Table S2.

The 12 selected publications are outlined in Table 1, which summarises the key characteristics of each source. Of the 12 sources, 11 publications reported primary research comprising program evaluations (*n* = 3) [20–22], mixed methods research (*n* = 4) [23–26], qualitative studies (*n* = 2) [27, 28] and synthesis of literature with key informant perspectives (*n* = 2) [29, 30]. The final publication was a practitioner report [31].

### Individual and family barriers

#### Financial cost

The financial cost of attaining and maintaining a licence was considered to be prohibitive by six publications [24–26, 28–30] The costs associated with licensing were frequently cited as the fees for tests and licences, which is problematic for those requiring several attempts at the Learner knowledge test (e.g. those with low literacy). There was also some overlap with meeting the requirements of the GDL scheme due to the cost of maintaining a suitable vehicle for supervisory driving practice, the cost of petrol and the cost of professional driving lessons.

#### Literacy issues

Low literacy, cited by 11 publications was the most widely reported barrier to licence participation [20–22, 24–31]. Primarily this was related to preparing for and passing the Learner driver knowledge test, however literacy was also deemed necessary for completing forms (e.g. proof of identification, licence application) and navigating the fines and debt system. Further, employment options can be limited for people with low literacy, thus not having access to a drivers licence was considered to be further reducing options for employment and economic participation.

#### Language

Having English as an additional language was cited as a barrier to licence participation in five publications [21, 23, 27, 28, 31]. This tended to be a more significant problem in remote communities where Aboriginal languages were primarily spoken and there was no access to interpreters for testing. One publication reported that in remote Northern Territory communities most people speak English as a second or third language [31]. One publication recommended that alternative testing options be considered including offering the option of verbal testing [23].

#### Lack of confidence

Insufficient confidence to navigate the licensing system was cited as a barrier to participation by four publications [22, 24, 25, 30]. There was variation in how the issue of confidence was framed, however across the publications it was typically described as a fear of failure, low self-esteem, and feelings of intimidation or shame, particularly in relation to licence testing. Confidence was ascribed to low literacy and a lack of cultural responsiveness in the system.

### Systemic barriers

#### Proof of identity documents

There were 10 publications citing accessing proof of identity documents to be a barrier to licensing [20–23, 25–27, 29–31]. These publications reported that eligibility to apply for a driver licence requires proof of identification in all Australian jurisdictions, however Aboriginal people often face complex barriers to obtaining the requisite documents including: having documents with multiple names, cost associated with applying for identification documents, literacy required to complete forms and access to service providers. An additional publication reported that accessing identity documents was a barrier, but was not a commonly cited barrier [24].

#### Meeting requirements of graduated driver licensing

There was widespread reporting that the supervised driving practice requirements of the GDL presents a major barrier for Aboriginal Learner drivers progressing to a provisional driver licence as reported by nine publications [20, 21, 23, 25–27, 29–31]. One publication reported that mandatory time periods on Learner licences are not conducive to running intensive driver training programs in remote communities that have transient populations [31].

#### Justice system

The justice system was identified by nine publications as a barrier to equitable participation in licensing, however this was described as a complex and multi-faceted issue that centres on Aboriginal people experiencing increased contact with the justice system and higher rates of incarceration due to licensing regulatory offences [20, 22–26, 28–30]. There were four main reasons that Aboriginal people were experiencing increased contact with the justice system that was precluding access to licensing: 1) fine default licensing sanctions due to inability to pay fines and/or state debt; 2) lack of diversionary options or programs for offenders; 3) unauthorised driving charges, which includes those who drive despite never having a licence and those who drive with a suspended or disqualified licence.

Five publications cited unauthorised driving as a major issue, which was attributed to low rates of licensed drivers, lack of understanding of the penalties and the need to travel by private car to access services, employment and meet cultural obligations [23, 24, 28–30]. Furthermore, two publications identified a detrimental cycle whereby those with existing licensing sanctions who drive unlicensed and are charged with secondary unauthorised driving face significant enforcement actions and likely incarceration [23, 26]. This risk of incarceration is increased due to a lack of diversionary options for magistrates to refer offenders; three publications recommend a diversion of resources from enforcement to delivering driver licensing services and increased support for existing diversionary programs e.g. Work and Development Orders [21, 23, 29].

#### Service provision

A lack of culturally responsive and aware service provision within the licensing system was considered to be a barrier to participation by seven publications [23, 24, 26–28, 30, 31]. Five publications identified a lack of local initiatives in communities to assist people to overcome barriers and access the licensing system [23, 26, 28, 30, 31]. Service provision was an issue in urban locations where service delivery was through state funded authorising agencies, and also in regional and remote communities where licensing services are frequently delivered by the police, which can be a strong deterrent for those who have had early negative experiences with police [27].

There were extensive issues cited with service provision in regional and remote locations with eight publications reporting barriers to licensing that were either specific or heightened for regional and remote communities [21–24, 27, 29–31]. One publication identified the lack of relevance of test content to drivers in remote communities and reported that certain traffic regulations were not applicable in remote contexts (e.g. roundabouts) [27]. There was general consensus that there was less service provision in regional and remote centres, and two publications asserted that accessing reliable and cost effective driving lessons or subsidised driver training programs (e.g. keys2drive^1^) is often not possible [21, 30].

### Cycle of licensing adversity

**Fig. 2:**
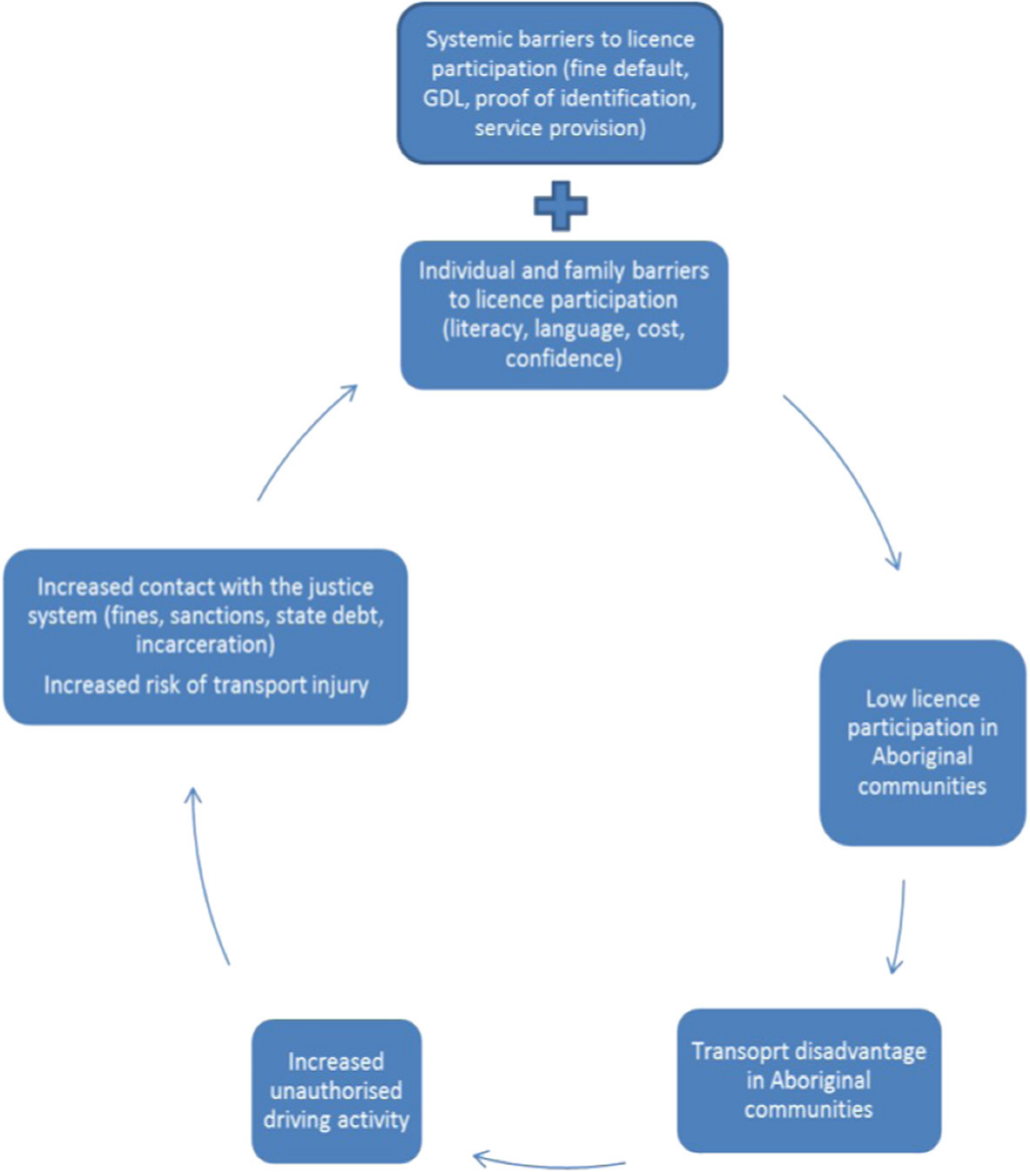
Cycle of Licensing Adversity

Across the publications it emerged that barriers to licensing are part of a cycle of adversity that contributes to disadvantage in Aboriginal communities; four publications described this cycle as self-perpetuating, particularly in relation to supervised driving practice, the fines enforcement system and unauthorised driving [21, 23, 26, 30]. Consequently, a cyclical relationship is presented between low rates of licence participation, transport disadvantage, increased risk of injury and increased contact with the justice system (Fig. 2). Individual and family barriers to licensing can be viewed within this cycle as both contributor and consequence of adversity within the licensing system.

## Discussion

This is the first systematic review of the literature on the barriers to licence participation among Aboriginal and Torres Strait Islander people across Australia. This review did not limit the included publications to a specific jurisdiction or type of publication as we sought to consider the impact of geographical, cultural and policy barriers. By looking at the evidence across multiple jurisdictions, it emerged that there are universal barriers to licensing impacting Aboriginal people across Australia. We were further able to conceptualise the barriers within a cycle of licensing adversity that depicts the relationships between licence participation, transport disadvantage and vulnerability to an inequitable system.

Within the cycle of licensing adversity, systemic barriers emerged as highly deterrent to equitable participation in licensing; the GDL, fines enforcement system and requisite identity documents are precluding vulnerable people from navigating the licensing and justice system. Consistent with this review, the issue of proof of identification has been previously identified as a barrier to employment, education and licensing [32]. While obtaining proof of identification is straightforward for many Australians, Aboriginal people can face specific barriers to accessing the requisite documents. Aboriginal people have a lower rate of birth registration, which presents a significant barrier to obtaining a birth certificate [32]. Access to identity documents can also be problematic where people may be known by more than one name, and may therefore have documents with multiple names or different spelling of the same name. There are also issues of identity that are experienced by people who have been displaced from their communities, in some cases they may not know their exact birth date and can have difficulty applying for legal identification documents [32]. Inability to access identification documents is highly prohibitive to driver licensing but also to accessing employment, education and housing; indeed it is a fundamental to equity and social inclusion to be able to access identification documents.

The Graduated Driver Licensing (GDL) schemes vary across jurisdictions in terms of the mandatory periods on Learner and Provisional licences and the requisite number of supervised driving hours to progress from a Learner to a Provisional licence [5]. Despite the variation, the GDL scheme is presenting a major barrier, primarily due to the requirements for supervised driving practice [6]. There is an interplay of systemic, individual and family factors that render many novice drivers in Aboriginal communities unable to meet the requirements of supervised driving practice. This review found it to be endemic in Aboriginal communities, whereby a shortage of licensed drivers able to act as supervisory drivers, lower rates of car ownership, the high cost of petrol and the high cost of professional driving lessons are proving to be frequently insurmountable barriers to meeting the supervised driving requirements of GDL. Publications citing this as the major barrier to licensing were most frequently based upon NSW data, where the GDL requires 120 h of supervised driving.

In terms of the justice system, fines are presenting a major barrier to participation; fines are issued for traffic and non-traffic offences including rail ticket violations, fisheries offences, failure to vote. The fines system is not means tested and there is a strong correlation whereby geographic regions with lower average incomes are more likely to have higher proportion of outstanding fines [26]. Consistent with this review, Golledge [11] asserts that those without means to pay fines are vulnerable to further enforcement actions due to fine default. Aboriginal people were identified as highly at risk of fine default, which is largely due to lower income but also relates to issues navigating the fines enforcement system and general lack of understanding of legal processes and requirements (e.g. court attendance) [26]. While automatic imprisonment for fine default has been abolished in all Australian jurisdictions, the frequent alternative is to impose licensing and/or vehicle registration sanctions on those who default on fine payments [11]. Essentially, this has seen vulnerable populations without a viable means to make payments having secondary sanctions imposed that prohibits maintaining or attaining a driver licence [11]. For example, in NSW, the rate of driver licences suspensions due to fine default are three times higher among Aboriginal people than the non-Aboriginal population [26]. For those without a driver licence, fine default results in sanctions that render them ineligible for applying for a licence until the fine is addressed.

Increased contact with the justice system resulting in licence disqualifications and sanctions has a ripple effect whereby vulnerable families have reduced options for transport, and subsequently reduced access to employment and essential services, which further marginalises those experiencing financial hardship and can be particularly devastating to those residing in regional and remote locations where travel by private car is essential [33]. Further, it is widely acknowledged that Aboriginal people have cultural and kinship obligations that can require travel and transporting family members; this review reinforces recommendations by Naylor [6] to implement an amnesty around licence disqualification in cases of extreme hardship due to Aboriginal kinship and cultural obligations.

Within the context of these policy barriers there is the issue of service provision. Firstly, access to relevant licensing agencies requires transport, and in regional and remote communities this is typically by private car. This presents a barrier particularly for unlicensed people in remote communities as the closest licensing agency is often a considerable distance, which adds to the costs and difficulty associated with accessing the agency. Further, there is an increased likelihood that the licensing services are delivered by the police in these locations, which can be a barrier for those who may have had previous negative experience with police and are not comfortable accessing the service [27]. There are strong recommendations for the government to fund community-based culturally responsive licensing service delivery and ensure that program sustainability is supported through robust evaluation [29]. Further, there is a need for community-based initiatives to have a high degree of cultural responsiveness and an understanding of community capacity building [3, 23, 28, 30].

Individual barriers were seen as both a contributor and consequence within the cycle of licensing adversity. Low rates of licence participation in Aboriginal communities contributes to transport disadvantage, with subsequent reduced access to essential services, employment, education and social opportunities [4, 33]. Transport disadvantage has been implicated in reduced health outcomes for Aboriginal people and unsafe road behaviours (e.g. driving unlicensed and vehicle overcrowding), which is related to increased contact with the justice system and increased risk of transport injury [4]. Ultimately the cycle of licensing adversity depicts the interrelationship between transport disadvantage and individual and family barriers to licensing within the context of systemic barriers to licensing.

In reviewing barriers to licensing, there is evidence that an endemic lack of access for Aboriginal people relates to financial hardship, unmet cultural needs and an inequitable system that is underservicing vulnerable populations. This review supports recommendations for targeting change at the systemic level within the authorising environment. This includes a review of proof of identification and fines enforcement policy, investment in diversionary programs, increased provision for verbal testing and subsidising the costs associated with licensing for people experiencing financial hardship. Access to licensing must also be addressed by service provision that is inclusive, responsive to the cultural needs of Aboriginal people and accessible to regional and remote communities.

While barriers to licensing in other Indigenous contexts globally (e.g. Native American, Canadian First Nations) has not been reported, there is evidence that Indigenous populations are over-represented in transport injury [34]. Further, evidence suggests that Indigenous populations experience significant transport disadvantage, which in New Zealand Maori populations has been described as ethnically mediated transport disadvantage [35]. Despite this, little is known about the role that licensing access may play as a protective factor against transport injury and transport disadvantage [34]. This study has provided insight into the barriers to driver licensing and participation in safe and legal driving among Aboriginal people in Australia; it is recommended that this approach could be used to explore barriers to licensing in other Indigenous populations globally.

Although a systematic search of the literature was conducted, there is potential that all relevant publications were not located, however the risk of this was minimised by extensively searching beyond electronic databases [36, 37]. Bias in article selection was minimised by having two authors independently screen articles, and there was a high level of agreement between the authors. The narrative synthesis of literature was deemed appropriate for the literature that has been published on Aboriginal driver licensing barriers, which is typically descriptive research rather than intervention research that is suited to meta-analysis. Moreover, the majority of the sources were from grey literature and six out of twelve articles were not suitable for quality appraisal. While there were considerable limitations with the quality of the publications, this reflects both the emerging status of Aboriginal driver licensing as a burgeoning focus of road safety and public health research and highlights the need for the conduct of robust evaluations and research in this area.

Research can support the recommendations for reform through conducting robust evaluations of policy and community initiatives. Designing effective initiatives to improve access for Aboriginal people must involve consultation with Aboriginal communities to determine the most culturally responsive approach that promotes equity, incorporates capacity building, local governance, Aboriginal leadership and is supported by inclusive policy [38, 39]. The impact of changes to policy should be investigated through the analysis of linked licensing, crash and hospitalisation data; this can only be conducted if accurate licensing data with Indigenous status is collected, which is currently only collected in NSW [3, 40, 41]. There is an urgent need to expand the collection of Indigenous status in licensing data and to promote identification and ensure a high level of data quality.

## Conclusion

This review highlights inherent inequity within the licensing and justice system that sees Aboriginal communities in Australia facing significant barriers to accessing a licence. Licensing adversity contributes to increased rates of transport injury and operates within a cycle of increased contact with the justice system and transport disadvantage in Aboriginal communities. Within this cycle, transport disadvantage impacts social inclusion through reduced access to employment, education, healthcare, social and cultural opportunities. While the need to improve the health and education of the Aboriginal population in Australia is well documented, this review explores the barriers to driver licensing, which is frequently overlooked as means to impact equity and social inclusion goals. Our review places barriers to licensing within the context of broader barriers to participation that Aboriginal people face including financial hardship, remoteness from service providers and unmet cultural needs. This review signifies a need to ensure equitable access to the licensing system by targeting reform at policy that inadvertently disadvantages Aboriginal people.

## Additional file

Additional file 1: Table S1. Search terms. **Table S2.** Explanations for exclusion. (DOCX 20 kb)

## Abbreviations

GDL: Graduate driver licensing
MMAT: Mixed methods appraisal tool
NSW: New South Wales

## Footnotes

1 The Keys2Drive program is a NSW government funded professional driving lesson with driving instructor and an additional accompanying supervisory driver.
