# Supplementary material for: Challenges to driver licensing participation for Aboriginal people in Australia: a systematic review of the literature
Source: Int J Equity Health. 2016 Aug 31;15(1):134. doi: 10.1186/s12939-016-0422-9 (PMC5007801; doi:10.1186/s12939-016-0422-9)
Supplement: Additional file 1: Table S1. — Search terms. Table S2. Explanations for exclusion. (DOCX 20 kb) [file 12939_2016_422_MOESM1_ESM.docx]

| Supplementary Table 1 Search terms |
| --- |

| 1 | Indigenous |
| --- | --- |
| 2 | Aborigin* |
| 3 | 1 or 2 |
| 4 | licen* |
| 5 | unlicen* |
| 6 | drive* |
| 7 | driving |
| 8 | road |
| 9 | transport* |
| 10 | 4 or 5 or 6 or 7 or 8 or 9 |
| 11 | safe* |
| 12 | program* |
| 13 | injur* |
| 14 | crash* |
| 15 | accident* |
| 16 | disadvantag* |
| 17 | 11 or 12 or 14 or 15 or 16 or 17 |
| 18 | 3 and 10 and 18 |

Supplementary Table 2 Explanations for exclusion

| Excluded record | Explanation – did not meet the following inclusion criteria |
| --- | --- |
| Clapham, Senserrick [1] | 5 |
| Currie and Senbergs [2] | 5 |
| Freethy [3] | 4 |
| Golledge [4] | 4 |
| Helps and Moller [5] | More recent report included |
| Hinchcliff, Holloway [6] | 4 |
| Department of Transport [7] | 4 |
| Ivers, Byrne [8] | 5 (Study protocol) |
| Ivers, Clapham [9] | 5 |
| Naylor [10] | 4 |
| Orenstein [11] | 5 |
| Pyne [12] | 5 |
| CARRS-Q [13] | 4 |
| Rosier and McDonald [14] | 5 |
| Senserrick [15] | 4 |
| Styles and Edmonston [16] | 5 |
| Transport for NSW [17] | 5 |
| Vermeulen [18] | 5 and methodology unclear |
| Vick [19] | 5 (focus is on community based educator model of driver training) |
| Vick and Avery [20] | 5 (focus on lifelong learning) |
| Zappia and Dell [21] | 4 |

Inclusion criteria:

| 1. From 2000 onwards |
| --- |
| 1. Australian context |
| 1. Full text available |
| 1. Specifically Aboriginal population |
| 1. Barriers to driver licensing focus |

Excluded References

1. Clapham, K., et al., *Understanding the extent and impact of Indigenous road trauma.* Injury, 2008. **39**(Supplement 5): p. S19-S23.

2. Currie, G. and Z. Senbergs, *Indigenous communities: Transport disadvantage and Aboriginal communities*, in *No Way To Go: Transport and Social Disadvantage in Australian Communities*, G. Currie, J. Stanley, and J. Stanely, Editors. 2007, Monash University ePress: Clayton, Victoria.

3. Freethy, C. *L2P – learner driver mentor program: extending driver licensing reach in disadvantaged communities*. in *Australasian College of Road Safety Conference*. 2012. Sydney, NSW.

4. Golledge, E., *Not such a fine thing! The impact of fines and the regulation of public space.* Parity, 2006. **19**(1): p. 58-59.

5. Helps, Y.L.M. and J. Moller, *Aboriginal People Travelling Well literature review: Driver licensing issues, seat restraint non-compliance, Aboriginal health, Aboriginal disability*. 2007, Adelaide: Research Centre for Injury Studies, Flinders University: Adelaide.

6. Hinchcliff, R., et al., *Barriers to obtaining a driver licence in regional and remote areas of Western NSW*, in *Australasian Road Safety Research, Policing & Education Conference, 12 – 14 November, Melbourne*. 2014.

7. Department of Transport, *Aboriginal eople and ethnic minority groups accessing our driver licensing services: Substantive equality framework assessment*, D.o. Transport, Editor. 2012, The Government of Western Australia: Perth.

8. Ivers, R., et al., *Development of a community based Aboriginal driver licensing service: the AstraZeneca Young Health Programme.*, in *Australasian College of Road Safety Conference*. 2012: Sydney, NSW.

9. Ivers, R., et al., *Collecting measures of Indigenous status in driver licencing data.* Australasian Epidemiologist, 2012. **19**(2): p. 9-10.

10. Naylor, B., *L-plates, logbooks and losing-out: Regulating for safety - or creating new criminals?* Alternative Law Journal, 2010. **35**(2): p. 94-98.

11. Orenstein, J., *The difficulties faced by Aboriginal Victorians in obtaining identification.* Indigenous Law Bulletin, 2008. **7**(8): p. 14-17.

12. Pyne, A., *Ten proposals to reduce indigenous over-representation in Northern Territory prisons*. 2012. p. 2-17.

13. CARRS-Q, *Rural and remote road safety research program : major recommendations*. 2008, Brisbane: Centre for Accident Research and Road Safety Brisbane.

14. Rosier, K. and M. McDonald, *The relationship between transport and disadvantage in Australia*. 2011, Australian Institute of Family Studies: Melbourne.

15. Senserrick, T.M., *Recent developments in young driver education, training and licensing in Australia.* Journal of Safety Research, 2007. **38**(2): p. 237-244.

16. Styles, T.O. and C. Edmonston, *Australian Indigenous road safety : 2005 update*. 2006, Canberra: Australian Transport Safety Bureau: Canberra.

17. Transport for NSW, *NSW Aboriginal Road Safety Action Plan 2014-2017*. 2014, Transport for NSW: Sydney.

18. Vermeulen, A.P., *Aboriginal road safety awareness and licensing program*. 2006, Kempsey: Kempsey Shire Council: Kempsey.

19. Vick, M., *Community education for social change: the development of driver licensing educational strategies in north Queensland Indigenous communities*, in *AARE National Conference*. 2007, Australian Association for Research in Education Fremantle, WA.

20. Vick, M. and D. Avery. *The Queensland Indigenous driver licensing program, community-based educators, and lifelong learning*. in *4th International Lifelong Learning Conference* 2006. Rockhampton, QLD.

21. Zappia, M. and C. Dell. *Learner driver mentor programs – what’s happening out there?* in *Australasian Road Safety Research, Policing and Education Conference*. 2009. Sydney, NSW.
